# Vinblastine and sulfinpyrazone export by the multidrug resistance protein MRP2 is associated with glutathione export

**DOI:** 10.1054/bjoc.2000.1262

**Published:** 2000-07-03

**Authors:** R Evers, M de Haas, R Sparidans, J Beijnen, P R Wielinga, J Lankelma, P Borst

**Affiliations:** Division of Molecular Biology and Center of Biomedical Genetics, The Netherlands Cancer Institute, Plesmanlaan 121, Amsterdam, CX, 1066, The Netherlands; Department of Pharmacy, Slotervaart Hospital, Louwesweg 6, Amsterdam, EC, 1066, The Netherlands; Department of Medical Oncology, Academic Hospital Vrije Universiteit, Room BR232, PO Box 7057, Amsterdam, MB, 1007, The Netherlands; Georg-Speyer-Haus, Paul Ehrlich straße 42–44, Frankfurt am, 60596, Germany

**Keywords:** multidrug resistance protein, polarized cell, glutathione, organic anion, drug transport, GS-X pump

## Abstract

The multidrug resistance proteins MRP1 and MRP2 are members of the same subfamily of ATP-binding cassette transporters. Besides organic molecules conjugated to negatively charged ligands, these proteins also transport cytotoxic drugs for which no negatively charged conjugates are known to exist. In polarized MDCKII cells, MRP1 routes to the lateral plasma membrane, and MRP2 to the apical plasma membrane. In these cells MRP1 transports daunorubicin, and MRP2 vinblastine; both transporters export reduced glutathione (GSH) into the medium. We demonstrate that glutathione transport in MDCKII-MRP1 cells is inhibited by the inhibitors of organic anion transporters sulfinpyrazone, indomethacin, probenecid and benzbromarone. In MDCKII-MRP2 cells, GSH export is stimulated by low concentrations of sulfinpyrazone or indomethacin, whereas export is inhibited down to control levels at high concentrations. We find that unmodified sulfinpyrazone is a substrate for MRP2, also at concentrations where GSH export is inhibited. We also show that GSH export in MDCKII-MRP2 cells increases in the presence of vinblastine, and that the stochiometry between drug and GSH exported is between two and three. Our data indicate that transport of sulfinpyrazone and vinblastine is associated with GSH export. However, at high sulfinpyrazone concentrations this compound is transported without GSH. Models of MRP action are discussed that could explain these results. © 2000 Cancer Research Campaign


British Journal of Cancer (2000) 83(3), 375–383
© 2000 Cancer Research Campaign
doi: 10.1054/ bjoc.2000.1262, available online at http://www.idealibrary.com on IDEAL®

### Vinblastine and sulfinpyrazone export by the multidrug resistance protein MRP2 is associated with glutathione export

**R Evers[1,4], M de Haas[1], R Sparidans[2], J Beijnen[2], PR Wielinga[3], J Lankelma[3] and P Borst[1]**

[1]Division of Molecular Biology and Center of Biomedical Genetics, The Netherlands Cancer Institute, Plesmanlaan 121, 1066 CX Amsterdam, The Netherlands; [2]Department of Pharmacy, Slotervaart Hospital, Louwesweg 6, 1066 EC Amsterdam, The Netherlands; [3]Academic Hospital Vrije Universiteit, Department of Medical Oncology, Room BR232, PO Box 7057, 1007 MB Amsterdam, The Netherlands; [4]Present address Georg-Speyer-Haus, Paul Ehrlich straße 42–44, 60596 Frankfurt am, Germany

**Summary** The multidrug resistance proteins MRP1 and MRP2 are members of the same subfamily of ATP-binding cassette transporters. Besides organic molecules conjugated to negatively charged ligands, these proteins also transport cytotoxic drugs for which no negatively charged conjugates are known to exist. In polarized MDCKII cells, MRP1 routes to the lateral plasma membrane, and MRP2 to the apical plasma membrane. In these cells MRP1 transports daunorubicin, and MRP2 vinblastine; both transporters export reduced glutathione (GSH) into the medium. We demonstrate that glutathione transport in MDCKII-MRP1 cells is inhibited by the inhibitors of organic anion transporters sulfinpyrazone, indomethacin, probenecid and benzbromarone. In MDCKII-MRP2 cells, GSH export is stimulated by low concentrations of sulfinpyrazone or indomethacin, whereas export is inhibited down to control levels at high concentrations. We find that unmodified sulfinpyrazone is a substrate for MRP2, also at concentrations where GSH export is inhibited. We also show that GSH export in MDCKII-MRP2 cells increases in the presence of vinblastine, and that the stochiometry between drug and GSH exported is between two and three. Our data indicate that transport of sulfinpyrazone and vinblastine is associated with GSH export. However, at high sulfinpyrazone concentrations this compound is transported without GSH. Models of MRP action are discussed that could explain these results. © 2000 Cancer Research Campaign

**Keywords** multidrug resistance protein; polarized cell; glutathione; organic anion; drug transport; GS-X pump

After selection for resistance to a single cytotoxic drug, tumour cells may become resistant against a whole range of drugs with different chemical structures and cellular targets, a phenomenon called multidrug resistance (MDR) (Higgins, 1992). Membrane proteins belonging to the ATP-binding cassette (ABC) family of transport proteins play a central role in resistance by actively decreasing the intracellular drug concentration. In humans, two members of the ABC-transporter family have been identified that can render tumour cells MDR: MDR1 P-glycoprotein (Pgp) (Gottesman et al, 1995), and the multidrug resistance protein (MRP1) (Cole and Deeley, 1998).

Overexpression of MRP1 is associated with an increased transport rate of a range of substrates that are conjugated to glutathione (GSH), glucuronide, or sulfate (Leier et al, 1994; Müller et al, 1994; Jedlitschky et al, 1997). Transporters with these characteristics are known as glutathione conjugate (GS-X) pumps (Ishikawa, 1992), or multispecific organic anion transporters (Oude Elferink et al, 1995). Besides organic anions MRP1 can also transport neutral and basic cytotoxic drugs not known to be conjugated to GSH or other negatively charged compounds (Cole et al, 1994; Zaman et al, 1994). Nevertheless, MRP1 requires the presence of intracellular glutathione for the transport of these drugs (Versantvoort et al, 1995; Zaman et al, 1995). The data available are most consistent with a model in which MRP1 co-transports cytotoxic drugs with GSH (Rappa et al, 1997; Loe et al, 1998).

Another ABC-transporter that shows homology to MRP1 is the canalicular multispecific organic anion transporter (cMOAT or MRP2) (Büchler et al, 1996; Paulusma et al, 1996; 1997; Ito et al, 1997). It is plausible that MRP2 could play a role in drug resistance, just as MRP1 does. Studies with mutant rats ($TR^-$/GY or EHBR) that lack the MRP2 protein in the hepatocanalicular membrane and transfection studies with *MRP2* cDNA showed that the substrate specificity of MRP2 is very similar to that of MRP1 (Jedlitschky et al, 1997), and suggested that MRP2 is able to transport several anticancer drugs (Chu et al, 1997; Masuda et al, 1997; Cui et al, 1999).

We previously made a set of Madin–Darby Canine Kidney (MDCKII)-derived cell lines stably expressing human *MRP1*, *MRP2* or *MDR1* cDNA. In these polarized cell lines MRP1 routes to the lateral plasma membrane, whereas MRP2 and MDR1 Pgp localize to the apical plasma membrane. MDCKII-MRP1 cells transport several glutathione *S*-conjugates to the basal side of a monolayer, and MDCKII-MRP2 cells to the apical side. Moreover, in these transfected cells MRP1 can transport the anthracycline daunorubicin and MRP2 the vinca alkaloid vinblastine (Bakos et al, 1998; Evers et al, 1998). Paulusma et al (1999) recently showed that MDCKII-MRP1 and MDCKII-MRP2 cells also export reduced glutathione in the absence of drugs, resulting in a lowered intracellular glutathione concentration in these cells.

*Received 10 January 2000*
*Revised 3 April 2000*
*Accepted 6 April 2000*

*Correspondence to:* P Borst

In this study, we present a more detailed investigation of GSH export by MDCKII-MRP1 and MDCKII-MRP2 cells.

### MATERIALS AND METHODS

### Materials

[$^3$H]Vinblastine (15 Ci mmol$^{-1}$) was obtained from Amersham International (Little Chalfont, UK). Acivicin, sulfinpyrazone, benzbromarone, indomethacin, and probenecid were obtained from Sigma Chemical Co. (St. Louis, MO, USA). GSH, NADPH, and glutathione reductase were from Boehringer (Mannheim, Germany).

### Cell lines

Polarized Madin–Darby Canine Kidney (MDCKII) cells stably expressing human *cMOAT*(*MRP2*), *MRP1*, or *MDR1* cDNA have been described before (Bakos et al, 1998; Evers et al, 1998). Previously, we described several MDCKII-cMOAT clones (Evers et al, 1998). In this study MDCKII-cMOAT17 cells were used, and in this paper we will refer to these cells as MDCKII-MRP2. Cells were cultured in DMEM with 10% foetal calf serum.

### GSH export assays

Cells were grown on microporous polycarbonate filters (3 µm pore size, 24.5 mm diameter, Transwell™ 3414; Costar Corp, Cambridge, MA, USA) as described before (Evers et al, 1998). Cells were cultured for 3 days. Medium was replaced daily and 2 h before starting the experiment. GSH-transport experiments were performed in Hanks Balanced Salt Solution (HBSS plus $CaCl_2$ (1.3 mM); 1 ml per compartment) in the presence of acivicin (0.5 mM) at 37°C and 5% $CO_2$. Samples were taken at the time-points indicated and total glutathione was determined according to the recycling method of Tietze (1969). Previous experiments have shown that less than 2% of total glutathione was in the oxidized (GSSG) from (Paulusma et al, 1999). Intracellular GSH was determined by dissolving the cells in 10% perchloric acid. Precipitated proteins were removed by centrifugation, and samples were neutralized by adding a solution containing MOPS (0.5 M) and KOH (5 M). Glutathione was determined as described above.

### Transport assays

[$^3$H]Vinblastine transport assays were carried out essentially as described (Evers et al, 1998), with the modification that HBSS medium was used instead of DMEM. Cells were seeded on polycarbonate filters (see above). The experiment was started by adding 2 ml of HBSS containing acivicin (0.5 mM), PSC833 (0.1 µM), and the indicated amount of drug to either the apical or basal compartment. Cells were incubated at 37°C in 5% $CO_2$, and 50 µl aliquots were taken at the time-points indicated. Radioactivity was measured as the fraction of total radioactivity added at the beginning of the experiment. Sulfinpyrazone transport was determined as for vinblastine. Samples were split into two halves: one half was used for measuring the glutathione concentration, the other for measuring sulfinpyrazone. Sulfinpyrazone concentrations were measured by HPLC. After a 2–20-fold dilution with water, samples (20 µl) were injected on a Symmetry C18 column (100 × 4.6 mm; 3.5 µm, Waters chromatography (Milford, MA, USA) at ambient temperature. The eluent comprised acetonitrile (55% v/v), water (44.9% v/v), and trifluoroacetic acid (0.1% v/v). The eluent flow was 1 ml min$^{-1}$ and UV-absorption at a wavelength of 240 nm was used as the detection method. The retention time of sulfinpyrazone was 2.5 min under these conditions. Dilutions of sulfinpyrazone in water were used as calibration standards.

**Table 1** Inhibition of GSH flux by MDCKII-derived clones by benzbromarone, probenecid, or indomethacin

| | Total GSH (nmoles per monolayer) | | | | | | | | |
|---|---|---|---|---|---|---|---|---|---|
| | MDCKII | | | MDCKII-MRP1 | | | MDCKII-MRP2 | | |
| | In medium | | | In medium | | | In medium | | |
| | Apical[a] | Basal[a] | Intracellular[a] | Apical[a] | Basal[a] | Intracellular[a] | Apical[a] | Basal[a] | Intracellular[a] |
| Control | 0.6 ± 0.1 | 1.1 ± 0.0 | 31.8 ± 2.9 | 0.7 ± 0.1 | 9.3 ± 1.5 | 3.8 ± 1.3 | 3.2 ± 0.2 | 0.8 ± 0.1 | 25.7 ± 3 |
| Probenecid (1 mM) | 0.2 ± 0.0 | 0.5 ± 0.0 | 37.4 ± 0.2 | 0.5 ± 0.0 | 9.1 ± 0.8 | 6.7 ± 0.5 | 1.9 ± 0.0 | 0.4 ± 0.0 | 25.4 ± 1.0 |
| (5 mM) | 0.0 ± 0.0 | 0.1 ± 0.0 | 41.3 ± 0.2 | 0.1 ± 0.0 | 0.9 ± 0.0 | 13.3 ± 1.4 | 1.3 ± 0.0 | 0.1 ± 0.0 | 30.4 ± 1.0 |
| Benzbromarone (5 µM) | 0.6 ± 0.0 | 0.7 ± 0.0 | 31.4 ± 1.5 | 0.4 ± 0.0 | 4.5 ± 0.0 | 5.6 ± 0.0 | 2.7 ± 0.3 | 0.5 ± 0.2 | 20.2 ± 0.4 |
| (25 µM) | 0.5 ± 0.1 | 0.6 ± 0.0 | 32.3 ± 3.3 | 0.1 ± 0.1 | 0.6 ± 0.0 | 10.5 ± 0.7 | 0.7 ± 0.0 | 0.1 ± 0.1 | 23.0 ± 1.5 |
| Indomethacin (25 µM) | 4.8 ± 0.1 | 1.1 ± 0.1 | 28.3 ± 0.9 | 0.6 ± 0.1 | 11.6 ± 0.6 | 0.8 ± 0.2 | 9.6 ± 0.2 | 0.7 ± 0.0 | 15.8 ± 0.3 |
| (50 µM) | 5.5 ± 0.2 | 1.0 ± 0.3 | 28.8 ± 2.2 | 0.5 ± 0.1 | 10.7 ± 1.0 | 1.2 ± 0.1 | 11.4 ± 0.0 | 0.7 ± 0.0 | 15.0 ± 1.2 |
| (100 µM) | 4.2 ± 0.0 | 0.6 ± 0.1 | 29.3 ± 3.6 | 0.4 ± 0.1 | 9.6 ± 1.2 | 2.0 ± 0.4 | 10.5 ± 0.2 | 0.6 ± 0.0 | 16.4 ± 0.0 |
| (200 µM) | 2.5 ± 0.3 | 0.6 ± 0.1 | 37.4 ± 0.2 | 0.2 ± 0.0 | 7.0 ± 0.3 | 4.3 ± 0.3 | 7.7 ± 0.1 | 0.5 ± 0.0 | 18.1 ± 1.4 |
| (400 µM) | 1.5 ± 0.2 | 0.4 ± 0.0 | 36.8 ± 0.7 | 0.3 ± 0.0 | 4.2 ± 0.2 | 8.3 ± 1.3 | 4.5 ± 0.1 | 0.3 ± 0.0 | 21.8 ± 0.2 |

[a]Numbers in the table represent amounts of GSH (in nmoles per monolayer) fluxed into the apical or basal medium at $t$ = 2 h, respectively. 'Intracellular' represents amounts of GSH measured in the cell lysates at $t$ = 2 h. Cells growing in a monolayer were incubated in the presence of the inhibitors indicated. Inhibitors were added to both the apical and basal compartment. At $t$ = 2 h samples were taken from both compartments and the GSH concentration was determined. To determine the total amount of intracellular glutathione at $t$ = 2 h, cells were lysed and the amount of glutathione in the cell lysates was determined. Values are means of one typical experiment performed in duplicate ± the variation between the two measurements.

 © 2000 Cancer Research Campaign

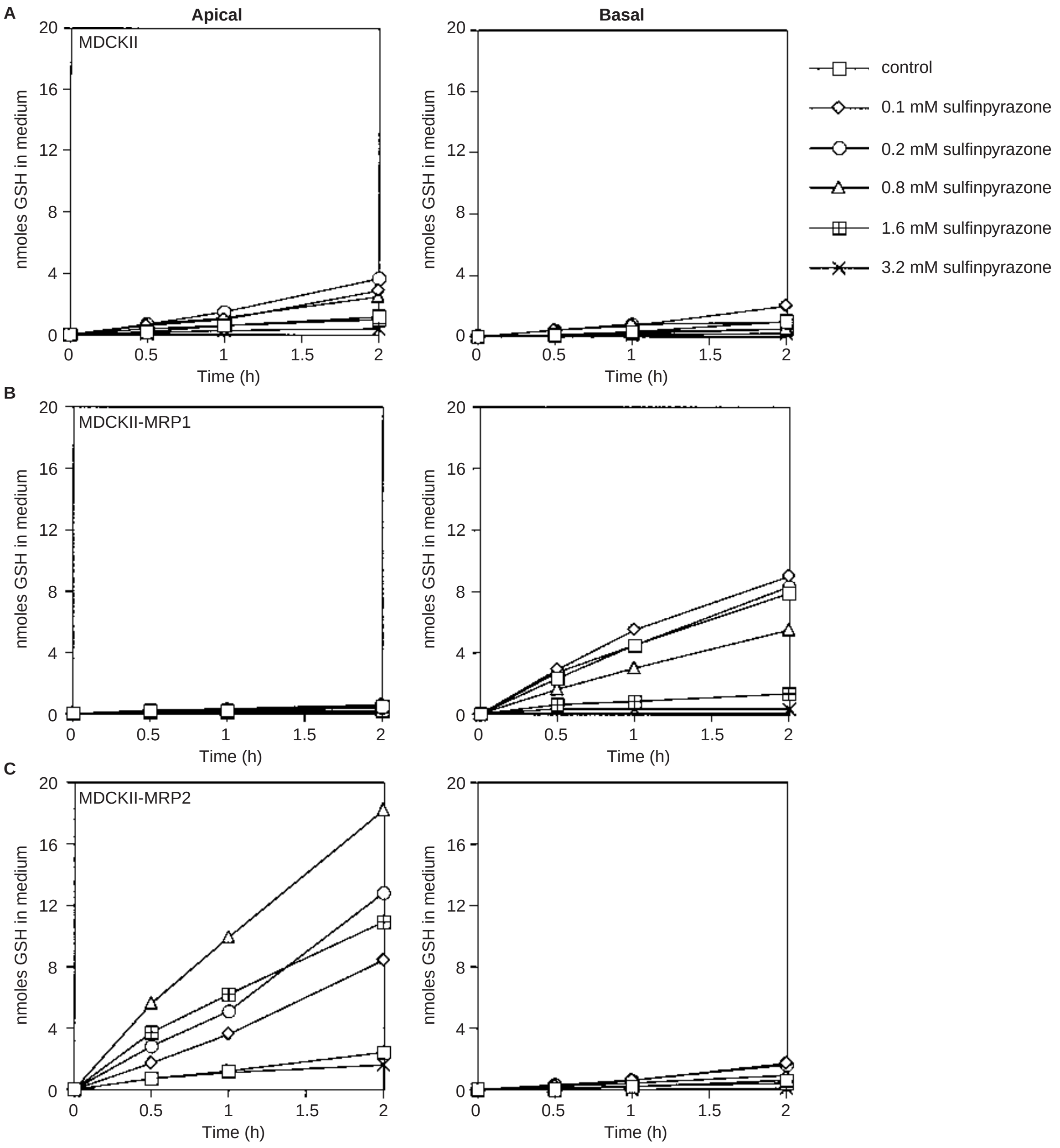

**Figure 1** Flux of GSH from MDCKII-derived clones in the presence of sulfinpyrazone. At $t = 0$ the indicated concentrations of sulfinpyrazone were applied to both the apical and basal side (in 1 ml HBSS per compartment) and the amount of glutathione appearing in the apical and basal compartment was determined. Samples were taken at $t = 30$, 60, and 120 min. Panels **A**, **B** and **C**: MDCKII, MDCKII-MRP1 and MDCKII-MRP2 cells were analysed, respectively. Experiments were performed in duplicate. Variations were within the size of the symbols

In experiments where the effect of cytotoxic drugs or inhibitors on GSH-transport was determined, compounds were added to both the apical and basal compartment at $t = 0$, unless indicated otherwise. Samples (50 µl) were taken at the time-points indicated.

### Calculations

Net flux at a particular substrate concentration was calculated by determining the net difference between the flux from basolateral to apical (ba), and vice versa (ab), in a transport experiment. The pump-mediated flux is controlled by the internal free substrate concentration. The latter is the result of both active and passive drug fluxes. As we observed a constant flux over time in our experiments, we concluded that the cellular loading time was relatively short. The intracellular concentration was therefore considered to be constant at the time-scale of the measurements.

The reasoning below shows that the net flux equals the pump-mediated flux for the simple case when: i) the pump-mediated flux follows Michaelis–Menten kinetics; ii) the substrate concentration is far below the $K_m$; and iii) the contribution of other transport proteins with very different transport characteristics is negligible.

© 2000 Cancer Research Campaign 

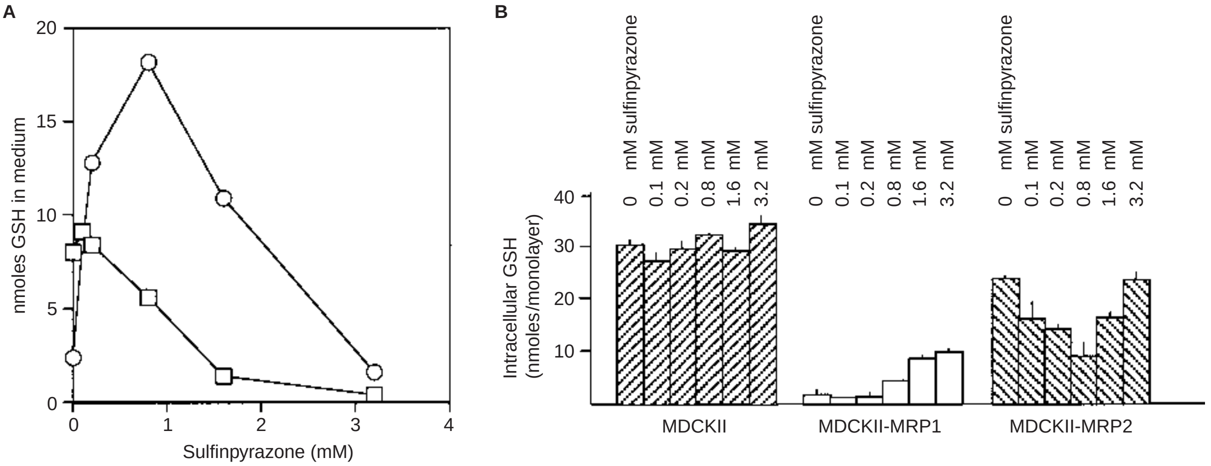

**Figure 2** Dose–response for sulfinpyrazone induced GSH flux and intracellular GSH levels. (**A**) Dose–response curve for sulfinpyrazone added to medium, and GSH exported into the basal (for MRP1; □) or apical (for MRP2; ○) medium, respectively. The GSH levels measured at $t = 2$ h were plotted (data derived from Figure 1). (**B**) Intracellular GSH levels (in nmoles per monolayer) in MDCKII-derived clones after a 2 h incubation in the presence of the indicated amounts of sulfinpyrazone

As:

total flux = flux through the cells + flux between the cells and:

net flux = flux through the $\text{cells}_{ba}$ – flux through the $\text{cells}_{ab}$

The term for flux between the cells cancels out in this subtraction as $C_a = C_b$, in both compartments, where $C_a$ = concentration in apical compartment and $C_b$ = concentration in basolateral compartment.

For the case of MRP2-mediated transport into the apical compartment, considering no backflux from the apical compartment and at quasi steady-state (at constant $C_i$):

$$\text{net flux} = C_{i,ba}\, k_a + C_{i,ba}\, V_{max}/K_m - C_{i,ab}\, k_b \qquad \text{(eq. 1)}$$

In which: $C_{i,ba}$ = intracellular free substrate concentration for flux from basal to apical transport (mol $L^{-1}$); $C_{i,ab}$ = intracellular free substrate concentration for flux from apical to basal transport (mol $l^{-1}$); $k_a$ = permeation coefficient of apical plasma membrane (l $min^{-1}$ $well^{-1}$); $k_b$ = permeation coefficient of basolateral plasma membrane (l $min^{-1}$ $well^{-1}$); $V_{max}$ = maximum pumping rate (mol $min^{-1}$ $well^{-1}$); $K_m$ = Michaelis constant for pumping (mol $l^{-1}$). Expressions for $C_{i,ba}$ and $C_{i,ab}$ can be found from the mass balances:

$$C_b k_b - C_{i,ba} k_b = C_{i,ba} k_a + C_{i,ba}\, V_{max}/K_m$$

and:

$$C_a k_a - C_{i,ab}\, V_{max}/K_m - C_{i,ab}\, k_a = C_{i,ab}\, k_b$$

Substitution of $C_{i,ab}$ and $C_{i,ba}$ into eq. 1 (at Ca = Cb) leads to:

net flux = $C_{i,ba}\, V_{max}/K_m$, which equals pump-mediated $\text{flux}_{ba}$.

### RESULTS

### Inhibition of GSH-transport

To assess whether classical inhibitors of organic anion transporters were able to inhibit the export of GSH in MDCKII-MRP1 and MDCKII-MRP2 cells, we measured GSH export in the presence of various concentrations of sulfinpyrazone, indomethacin, probenecid, or benzbromarone. Cells were grown in a monolayer on a porous membrane, enabling the measurement of polarized transport to both the apical and basolateral side of the cell monolayer. GSH export in the presence of sulfinpyrazone is shown in Figure 1; data obtained with the other compounds are summarized in Table 1. At relatively high concentrations a clear inhibition of the GSH export to the basolateral side of the cell monolayer was observed with all compounds in the MDCKII-MRP1 cells. Apical export in MDCKII-MRP2 cells was completely inhibited by benzbromarone, and partially by probenecid, but not by sulfinpyrazone or indomethacin. Unexpectedly, sulfinpyrazone and indomethacin strongly stimulated apical GSH export in MDCKII-MRP2 cells at relatively low concentrations (Figure 1C, Figure 2A, and Table 1). At higher concentrations, export of GSH started decreasing, but export could not be inhibited to the level observed in wild-type cells. Low concentrations of sulfinpyrazone also weakly stimulated the apical GSH flux by MDCKII wild-type cells (Figure 1A). This is probably caused by the presence of canine MRP2 in these cells (*see* Evers et al, 1998). Basolateral GSH export in the MDCKII-MRP1 cells was slightly stimulated by sulfinpyrazone and indomethacin. After incubation in the presence of inhibitors the intracellular glutathione levels roughly correlated in an inverse manner with the amounts of GSH that were exported (see Table 1 and Figure 2B). The inhibitory effect of high concentrations of sulfinpyrazone on the export of GSH was not simply due to a toxic effect as MDR1-mediated vinblastine transport by MDCKII-MDR1 cells was not affected (data not shown).

### Sulfinpyrazone transport by MDCKII derived clones

A possible explanation for the stimulatory effect of sulfinpyrazone on GSH export is that both compounds are co-transported with positive cooperativity. To test this hypothesis, we studied vectorial transport of sulfinpyrazone in MDCKII and MDCKII-MRP2 cells. Various concentrations of sulfinpyrazone were added to either the apical or basolateral side of the cell monolayer, and the

 *© 2000 Cancer Research Campaign*

**Table 2** Calculated flux of sulfinpyrazone through monolayers of MDCKII and MDCKII-MRP2 cells after 2 h. GSH concentrations were determined in the same samples

| | | GSH (nmoles per monolayer) | | | | Sulfinpyrazone (nmoles per monolayer) | | | |
|---|---|---|---|---|---|---|---|---|---|
| | | Sulfinpyrazone apical[a] | | Sulfinpyrazone basolateral[b] | | Sulfinpyrazone apical[a] | Sulfinpyrazone basolateral[b] | | |
| Cell line | Sulfinpyrazone concentration (mM) | Apical[d] | Basolateral[d] | Apical[d] | Basolateral[d] | Apical[d] | Basolateral [d] | Net flux of sulfinpyrazone[c] | Sulfinpyrazone/ apical GSH |
| MDCKII | 0 | 3.6 | 1.7 | 3.6 | 1.7 | – | – | – | – |
| | 0.2 | 4.0 | 2.5 | 5.0 | 2.4 | 20.5 | 7.4 | 13 | 2.6 |
| | 0.8 | 4.0 | 2.4 | 3.2 | 2.1 | 60 | 32 | 28 | 8.8 |
| | 1.6 | 6.0 | 1.8 | 2.5 | 1.8 | 136 | 75 | 61 | 24 |
| | 3.2 | 3.0 | 0.8 | 1.4 | 0.8 | 363 | 311 | 52 | 37 |
| MDCKII-MRP2 | 0 | 2.4 | 0.6 | 2.4 | 0.6 | – | – | – | – |
| | 0.2 | 7.0 | 1.4 | 12.7 | 1.6 | 44.6 | 4.8 | 40 | 3.1 |
| | 0.8 | 16.2 | 1.9 | 17.6 | 1.3 | 157 | 23.5 | 133 | 7.6 |
| | 1.6 | 18.2 | 1.6 | 10.1 | 0.7 | 255 | 52.2 | 203 | 20 |
| | 3.2 | 10.9 | 1.0 | 2.7 | 0.3 | 486 | 240 | 246 | 91 |

[a]Sulfinpyrazone was added to the apical compartment. [b]Sulfinpyrazone was added to the basal compartment. Apical GSH values from this experiment were used to calculate the ratio sulfinpyrazone/GSH. [c]Net transport is calculated as translocation from basal to apical minus translocation from apical to basal (data derived from Figure 3). [d]Numbers in the table represent the amounts of GSH that were measured in the apical or basal medium, respectively, at $t = 2$ h.

**Table 3** Vinblastine and GSH flux at various vinblastine concentrations in MDCKII and MDCKII-MRP2 cells

| | | Vinblastine transport | | Apical GSH flux | |
|---|---|---|---|---|---|
| Cell line | Vinblastine (μM) | % | Net flux (nmoles) | nmoles | Ratio vinblastine/GSH |
| MDCKII | 0 | – | – | 0.6 | – |
| | 10 | 4.1 | 0.8 | 0.9 | 0.9 |
| | 20 | 4.6 | 1.6 | 1.0 | 1.6 |
| | 30 | 4.2 | 2.6 | 1.3 | 1.8 |
| | 50 | 4.3 | 4.1 | 1.6 | 2.5 |
| MDCKII-MRP2 | 0 | – | – | 1.2 | – |
| | 10 | 22.6 | 4.6 | 2.2 | 2.1 |
| | 20 | 25.5 | 10.4 | 3.4 | 3.1 |
| | 30 | 22.7 | 13.6 | 5.9 | 2.3 |
| | 50 | 22.5 | 22.4 | 9.4 | 2.4 |

Cells growing in double-well plates were incubated in HBSS (2 ml per compartment) with the concentrations of [$^3$H]vinblastine indicated in the presence of PSC833 (0.1 μM). [$^3$H]Vinblastine was applied either in the apical or basal compartment, and the percentage of radioactivity appearing in the opposite compartment was determined. The % of vinblastine transported was determined by subtracting the amount of vinblastine translocated from apical to basal from the translocation from basal to apical. From this value the net vinblastine transport was calculated. The amount of GSH exported was determined in a parallel sample in which vinblastine was added to the basal compartment. Samples were taken at $t = 2$ h. Values represent means of an experiment performed in duplicate.

© 2000 Cancer Research Campaign

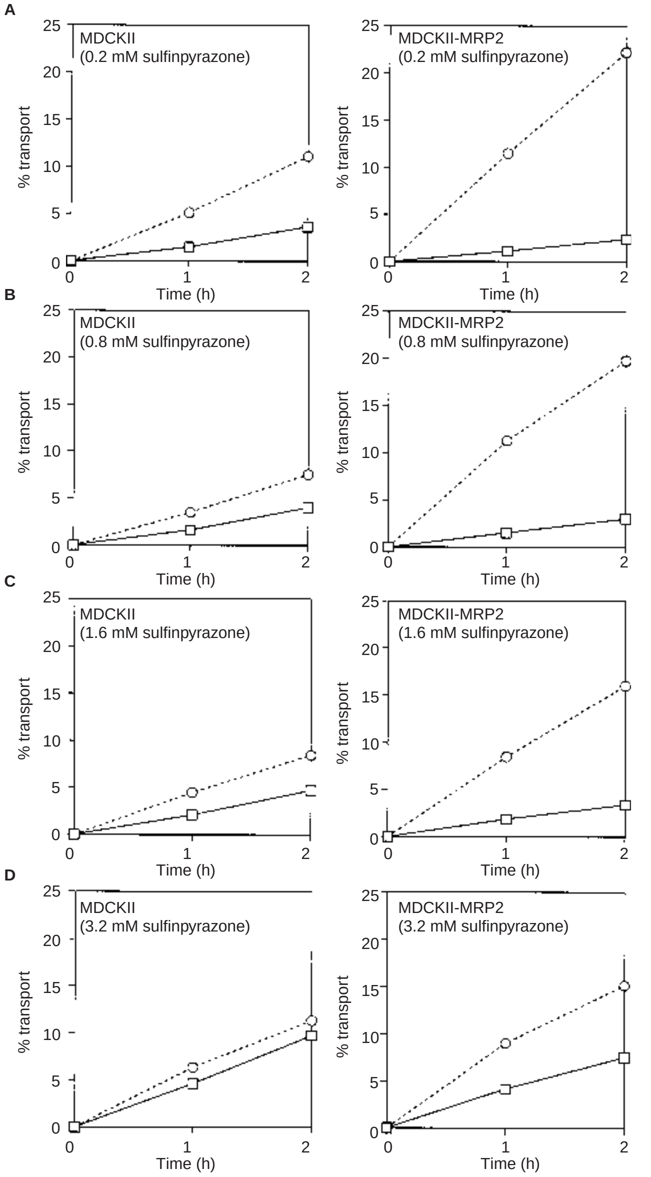

**Figure 3** Transepithelial flux of sulfinpyrazone by MDCKII and MDCKII-MRP2 monolayers. (**A**) At $t = 0$ sulfinpyrazone (0.2 mM) was applied to either the apical or basal compartment, and the percentage of sulfinpyrazone appearing in the opposite compartment was determined by HPLC analysis. Transport is presented as the fraction of sulfinpyrazone added at the beginning of the experiment appearing in the opposite compartment. Samples were taken at $t = 1$ and 2 h. (**B, C, D**) Same as **A**, but with 0.8, 1.6, or 3.2 mM sulfinpyrazone, respectively. Dashed line and ○: translocation from the basal to the apical compartment. Continuous line and □: translocation from the apical to the basal compartment. Experiments were performed in duplicate accumulation of sulfinpyrazone appearing in the opposite compartment was followed by a quantitative HPLC method (Figure 3). The total amount of sulfinpyrazone in the transport wells, as measured in the samples from both compartments, did not decrease in the course of the experiment, indicating that sulfinpyrazone was not metabolized by these cells (data not shown). The apical flux was higher than the basolateral flux in wild-type cells, suggesting that the MDCKII cells contain an endogenous apical transport mechanism for sulfinpyrazone. At all concentrations tested the amount of sulfinpyrazone translocated to the apical compartment was substantially higher in MDCKII-MRP2 than in wild-type cells, resulting in a clearly higher net flux of sulfinpyrazone in MDCKII-MRP2 cells (Table 2). Between 0.2 mM and 0.8 mM sulfinpyrazone net flux increased 3.3-fold, whereas between 0.8 mM and 3.2 mM this increase was only 1.8-fold, indicating that at sulfinpyrazone concentrations above 0.8 mM, transport became saturated. We conclude from these experiments that MRP2 causes vectorial transport of sulfinpyrazone.

To assess the correlation between the amounts of sulfinpyrazone and GSH transported by the MDCKII and MDCKII-MRP2 cells, GSH concentrations were measured in the same samples that were used for the sulfinpyrazone determinations. A complication of this experiment was that the amount of sulfinpyrazone required to inhibit GSH export was higher if sulfinpyrazone was added to the apical than to the basal compartment (Table 2, and *see* Wielinga et al, 1999). For further calculations the amounts of GSH exported to the apical compartment after adding sulfinpyrazone to the basal compartment were used. After 2 h the ratio between the amount of sulfinpyrazone and GSH transported in the MDCKII-MRP2 cells increased from 3.1 at 0.2 mM sulfinpyrazone to 91 at 3.2 mM (Table 2). These data suggest that at low sulfinpyrazone concentrations, where transport is most efficient, sulfinpyrazone transport is associated with GSH export, but that at high sulfinpyrazone concentrations this compound is transported without GSH.

We note in passing that the basal GSH excretion of MDCKII parental cells and MRP2 transfectants varies somewhat depending on the time cells were kept in culture (see e.g. Table 2). These variations did not affect the drug-induced GSH stimulation.

### Vinblastine-induced glutathione transport in MDCKII-MRP2 cells

We have previously shown that vinblastine is transported to the apical side of MDCKII-MRP2 cells (Evers et al, 1998). As MDCKII cells contain a relatively high concentration of MDR1 Pgp in the apical membrane (Horio et al, 1989), these experiments were performed in the presence of a low concentration of the Pgp inhibitor PSC833 (0.1 μM). This concentration of PSC833 does not significantly affect MRP2, whereas the endogenous MDR1 Pgp activity is efficiently blocked. We investigated whether vinblastine transport had an effect on the amount of GSH exported by MDCKII-MRP2 and wild-type cells. Figure 4 shows that GSH export to the apical side of a MDCKII-MRP2 cell monolayer strongly increased with increasing concentrations of vinblastine. This was not simply due to a toxic effect brought about by the relatively high concentrations of vinblastine used, as this increase was not observed in MDCKII-MRP1 or wild-type cells (Figure 4A and data not shown). To investigate the ratio between GSH and vinblastine transported by MDCKII-MRP2 cells, monolayers were incubated in the presence of various concentrations of [$^{3}$H]vinblastine in either the apical or basal compartment and radioactivity appearing in the opposite compartment was measured. In a parallel sample, the concentration of GSH was measured. Table 3 shows that an increase in vinblastine flux was accompanied with a concomitant increase in GSH export. The ratio vinblastine/GSH transported was between two and three at the concentrations tested.

We have shown before that daunorubicin is not transported by MDCKII-MRP2 cells (Evers et al, 1998), whereas it is transported

 *© 2000 Cancer Research Campaign*

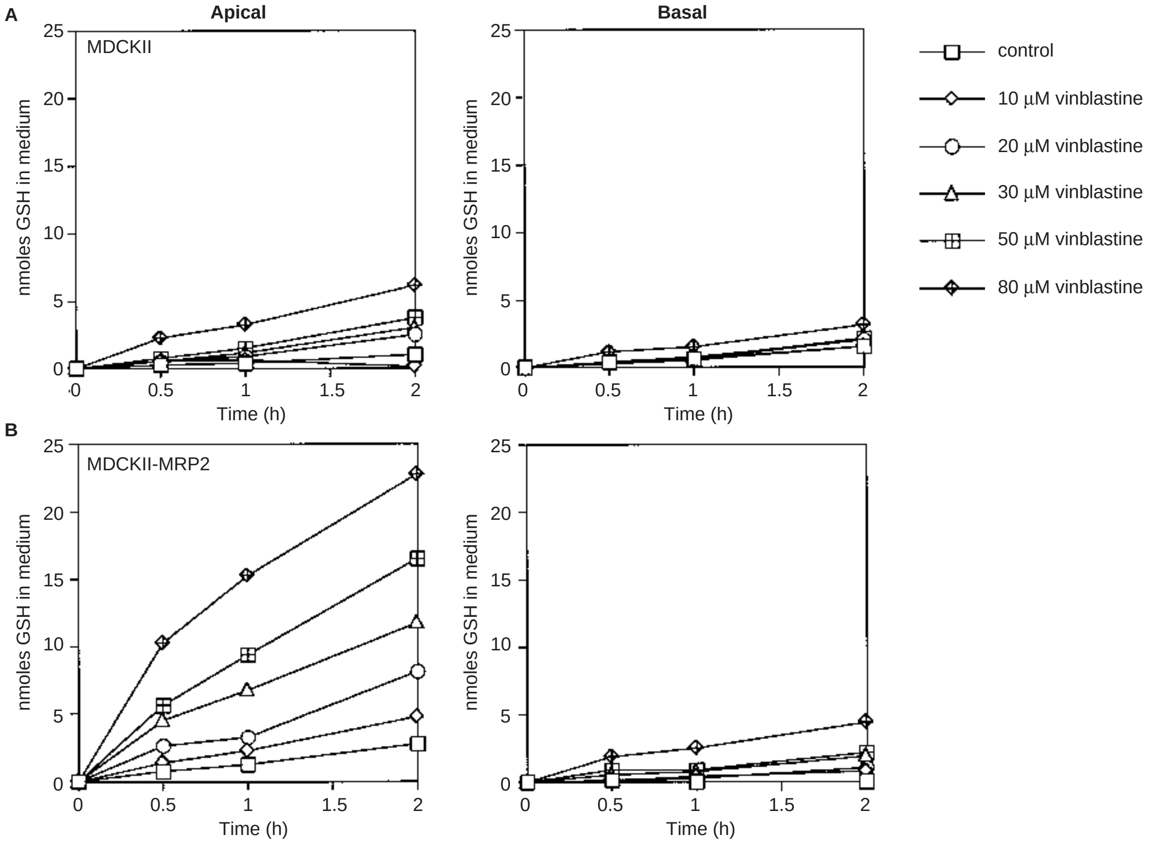

**Figure 4** Flux of GSH from MDCKII-derived clones in the presence of vinblastine. At $t = 0$ the indicated concentrations of vinblastine were applied to both the apical and basal compartment, and the amount of glutathione appearing in both compartments was determined. Experiments were performed in the presence of PSC833 (0.1 µM) and acivicin (0.5 mM). Samples were taken at $t = 30$, 60, and 120 min. (**A**) MDCKII cells, (**B**) MDCKII-MRP2 cells. Experiments were performed in duplicate at low rate by MDCKII-MRP1 cells (Bakos et al, 1998). We measured the GSH flux in the presence of various concentrations of daunorubicin (2–50 µM). No significant effect was observed on GSH transport under these conditions (data not shown).

### DISCUSSION

In this paper we show that GSH export in MDCKII-MRP1 cells is blocked by the inhibitors of organic anion transporters sulfinpyrazone, benzbromarone and probenecid, whereas export is partially inhibited by indomethacin. These results are in agreement with earlier findings that these compounds are inhibitors of drug transport by MRP1 (Feller et al, 1995; Evers et al, 1996; Holló et al, 1996; Heijn et al, 1997). Benzbromarone also blocked GSH export in the MDCKII-MRP2 cells, and probenecid gave a partial inhibition. Previously, we found that sulfinpyrazone and indomethacin had a stimulatory rather than an inhibitory effect on the transport of *S*-(2,4-dinitrophenyl)-glutathione (DNP-GS) by MDCKII-MRP2 cells (Evers et al, 1998). Here we show that low concentrations of sulfinpyrazone and indomethacin also stimulate GSH export in MDCKII-MRP2 cells, whereas high concentrations inhibit. As GSH export by MDCKII-MRP2 cells increases in the presence of vinblastine as well, our results suggest that transport of these drugs is associated with export of GSH. Transport experiments with a concentration range of sulfinpyrazone indicate that the transport mechanism may be more complex than obligatory co-transport of drug with GSH, as unmodified sulfinpyrazone is also transported by MDCKII-MRP2 cells at concentrations where GSH export is inhibited (Table 2). These data strongly suggest that sulfinpyrazone is also transported without GSH.

A critical point in the interpretation of our transport data with vinblastine is the assumption that this compound is not conjugated to GSH or other negatively charged groups. The evidence available suggests that such conjugates do not exist for anthracyclines or vinca alkaloids (Tew, 1994). Zaman et al (1995) analysed the medium from *MRP1*-overexpressing cells incubated in the presence of vincristine or daunorubicin and found that all drug recovered was in the unmodified form. The simplest interpretation of our data is that vinblastine is transported as free drug.

Several lines of evidence indicate that GSH is required for the transport of cytotoxic drugs by MRP1 (Versantvoort et al, 1995; Zaman et al, 1995; Loe et al, 1996; 1998; Rappa et al, 1997). However, in none of these experiments the stochiometry between drug and GSH transport was determined. In the MDCKII-MRP2 cells we observed that the GSH flux was dependent on the vinblastine concentration. The exact stochiometry between the amount of vinblastine and GSH transported is difficult to calculate. Vinblastine is a relatively hydrophobic compound that diffuses

*© 2000 Cancer Research Campaign*

easily over the plasma membrane and the determination of net flux therefore requires substantial correction factors. Nevertheless, our experiments strongly suggest that both compounds are co-transported (Table 3).

The stimulation of GSH export by sulfinpyrazone and vinblastine makes it unlikely that GSH is required to allosterically regulate the transport of drugs by MRP2. We propose a working model in which MRP2 has two drug-binding sites: one with a relatively high affinity for GSH (G-site) and a low affinity for drug, and one with a relatively high affinity for drug and a low affinity for GSH (D-site). The presence of a GSH-binding site is not unlikely as Taguchi et al (1997) have shown by vanadate trapping experiments that GSH can bind to MRP1, and Bakos et al (2000) have found GSH-induced ATP-ase activity in MRP1- and MRP2-containing membrane vesicles. We think that both binding sites are occupied by GSH in the absence of drugs, resulting in a slow export of GSH (Paulusma et al, 1999). We cannot rule out the alternative, however, that GSH export in the absence of added drug is accompanied by a not as yet identified intracellular metabolite that is binding to the D-site and is co-transported with GSH. We propose that at low drug concentrations the G-site remains occupied by GSH and the D-site becomes occupied by drug, resulting in co-transport of both compounds. We speculate that the two sites show positive cooperativity as we observe stimulation of GSH transport in the presence of the organic anions sulfinpyrazone and indomethacin. This may also explain why sulfinpyrazone is able to stimulate DNP-GS export in MDCKII-MRP2 cells (Evers et al, 1998). At high drug concentrations some (negatively charged) drugs appear to be able to occupy both the G- and D-site. In the case of sulfinpyrazone this results in transport of drug alone by MRP2. That sulfinpyrazone is directly transported by MRP2 and not by another fortuitously activated canine transporter is further substantiated by the finding that sulfinpyrazone inhibits the vinblastine flux by MDCKII-MRP2 cells (data not shown). MRP1 and MRP2 do not require free GSH for the transport of compounds that are conjugated to glutathione, glucuronide or sulfate (Jedlitschky et al, 1996; 1997; Evers et al, 1998; Ito et al, 1998). We suggest that such substrates have a relatively high affinity for both the G- and D-site and are therefore transported efficiently without requiring GSH or stimulating GSH export.

For MRP1, Heijn et al (1997) found, in uptake experiments with MRP1-containing vesicles, that DNP-GS uptake was non-competitively inhibited by daunorubicin, whereas uptake of GSSG was competitively inhibited. These authors proposed a substrate-binding site on MRP1 that consists of a pocket in which both daunorubicin and DNP-GS or GSSG bind in random order to different, only partly overlapping, sites. In this pocket, binding of a second compound is influenced by the compound that was bound first. Detailed in vitro experiments with MRP1- and MRP2-containing vesicles are required to critically test such models.

Whether MRP2 plays a role in clinical drug resistance remains to be seen. However, the availability of efficient inhibitors for this transporter would be helpful to address this question. The work presented here indicates that known inhibitors of organic anion transporters are either poor blockers of MRP2 or have the unexpected effect that they stimulate GSH export, something that may induce secondary reactions in cells. Induction of GSH export by sulfinpyrazone is not restricted to MDCKII-MRP2 cells, as we also observe it in other cell lines stably expressing *MRP2* (M Kool and PB, manuscript in preparation), indicating that the observations described here are not cell-line-dependent.

### ACKNOWLEDGEMENTS

We thank J Wijnholds, Z Holló, M Kool, and N Zelcer for critical comments on the manuscript. This work has been supported by Dutch Cancer Society Research Grant NK1 98–1794 (to PB and Dr Frank Baas).

### REFERENCES

Bakos E, Evers R, Szákacs G, Tusnády GE, Welker E, Szabó K, de Haas M, van Deemter L, Borst P, Váradi A and Sarkadi B (1998) Functional multidrug resistance protein (MRP1) lacking the N-terminal transmembrane domain. *J Biol Chem* **273**: 32167–32175

Bakos E, Evers R, Sinko E, Varadi A, Borst P and Sarkadi B (2000) Interaction of the human multidrug resistance proteins MRP1 and MRP2 with organic anions. *Mol Pharmacol* **57**: 760–768

Büchler M, König J, Brom M, Kartenbeck J, Spring H, Horie T and Keppler D (1996) cDNA cloning of the hepatocyte canalicular isoform of the multidrug resistance protein, cMRP, reveals a novel conjugate export pump deficient in hyperbilirubinemic rats. *J Biol Chem* **271**: 15091–15098

Chu XY, Kato Y and Sugiyama Y (1997) Multiplicity of biliary excretion mechanism for irinotecan, CPT11, and its metabolites in rats. *Cancer Res* **57**: 1934–1938

Cole SPC and Deeley RG (1998) Multidrug resistance mediated by the ATP-binding cassette transporter protein MRP. *Bioassays* **20**: 931–940

Cole SPC, Sparks KE, Fraser K, Loe DW, Grant CE, Wilson GM and Deeley RG (1994) Pharmacological characterization of multidrug resistant MRP-transfected human tumour cells. *Cancer Res* **54**: 5902–5910

Cui Y, Buchholz JK, Spring H, Leier I and Keppler D (1999) Drug resistance and ATP dependent conjugate transport mediated by the apical multidrug resistance protein, MRP2, permanently expressed in human an canine cells. *Mol Pharmacol* **55**: 929–937

Evers R, Zaman GJ, van Deemter L, Jansen H, Calafat J, Oomen LC, Oude Elferink RP, Borst P and Schinkel AH (1996) Basolateral localization and export activity of the human multidrug resistance-associated protein in polarized pig kidney cells. *J Clin Invest* **97**: 1211–1218

Evers R, Kool M, van Deemter L, Janssen H, Calafat J, Oomen LCJM, Paulusma CC, Oude Elferink RPJ, Baas F, Schinkel AH and Borst P (1998) Drug export activity of the human canalicular multispecific organic anion transporter in polarized kidney MDCK cells expressing cMOAT (MRP2) cDNA. *J Clin Invest* **101**: 1310–1319

Feller N, Broxterman HJ, Währer DCR and Pinedo HM (1995) ATP-dependent efflux of calcein by the multidrug resistance protein (MRP): no inhibition by intracellular glutathione depletion. *FEBS Lett* **368**: 385–388

Gottesman MM, Hrycyna CA, Schoenlein PV, Germann UA and Pastan I (1995) Genetic analysis of the multidrug transporter. *Annu Rev Genet* **29**: 607–649

Heijn M, Hooijberg JH, Scheffer GL, Szabo G, Westerhoff HV and Lankelma J (1997) Anthracyclines modulate multidrug resistance protein (MRP) mediated organic anion transport. *Biochem Biophys Acta* **1326**: 12–22

Higgins CF (1992) ABC-transporters: from microorganisms to man. *Annu Rev Cell Biol* **8**: 67–113

Holló Z, Homolya L, Hegedûs T and Sarkadi B (1996) Transport properties of the multidrug resistance-associated protein (MRP) in human tumour cells. *FEBS Lett* **383**: 99–104

Horio M, Chin K-V, Currier SJ, Goldenberg S, Williams C, Pastan I and Gottesman MM (1989) Transepithelial transport of drugs by the multidrug transporter in cultured Madin-Darby canine kidney cell epithelia. *J Biol Chem* **264**: 14880–14884

Ishikawa T (1992) The ATP-dependent glutathione S-conjugate pump. *Trends Biochem Sci* **17**: 463–468

Ito K, Suzuki H, Hirohashi T, Kume K, Shimizu T and Sugiyama Y (1997) Molecular cloning of canalicular multispecific organic anion transporter defective in EHBR. *Am J Physiol* **272**: G16–22

Ito K, Suzuki H, Hirohashi T, Kume K, Shimizu T and Sugiyama Y (1998) Functional analysis of a canalicular multispecific organic anion transporter cloned from rat liver. *J Biol Chem* **273**: 1684–1688

Jedlitschky G, Leier I, Buchholz U, Barnouin K, Kurz G and Keppler D (1996) Transport of glutathione, glucuronate, and sulphate conjugate by the MRP gene-encoded conjugate export pump. *Cancer Res* **56**: 988–994

Jedlitschky G, Leier I, Buchholz U, Hummel-Eisenbeiss J, Burchel B and Keppler D (1997) ATP-dependent transport of bilirubin glucuronides by the multidrug resistance protein MRP1 and its hepatocyte canalicular isoform MRP2. *Biochem J* **327**: 305–310

© 2000 Cancer Research Campaign

Leier I, Jedlitschky G, Buchholz U, Cole SPC, Deeley RG and Keppler D (1994) The MRP gene encodes an ATP-dependent export pump for leukotriene C4 and structurally related conjugates. *J Biol Chem* **269**: 27807–27810
Loe DW, Almquist KC, Cole SPC and Deeley RG (1996) Multidrug resistance protein (MRP)-mediated transport of leukotriene C4 and chemotherapeutic agents in membrane vesicles. *J Biol Chem* **271**: 9683–9689
Loe DW, Deeley RG and Cole SPC (1998) Characterization of vincristine transport by the M(r) 190 000 multidrug resistance protein (MRP): evidence for cotransport with reduced glutathione. *Cancer Res* **58**: 5130–5136
Masuda M, I'izuka Y, Yamazaki M, Nishigaki R, Kato Y, Ni'inuma K, Suzuki H and Sugiyama Y (1997) Methotrexate is excreted into bile by the canalicular multispecific organic anion transporter in rats. *Cancer Res* **57**: 3506–3510
Müller M, Meijer C, Zaman GJR, Borst P, Scheper RJ, Mulder NH, de Vries EGE and Jansen PLM (1994) Overexpression of the gene encoding the multidrug resistance-associated protein results in increased ATP-dependent glutathione *S*-conjugate transport. *Proc Natl Acad Sci USA* **91**: 13033–13037
Oude Elferink RPJ, Meijer DKF, Kuipers F, Jansen PLM, Groen AK and Groothuis GMM (1995) Hepatobiliary secretion of organic compounds, molecular mechanism of membrane transport. *Biochem Biophys Acta* **1241**: 215–268
Paulusma C, Bosma PJ, Zaman GJR, Bakker CTM, Otter M, Scheffer GL, Scheper RJ, Borst P and Oude Elferink RPJ (1996) Congenital jaundice in rats with a mutation in a multidrug resistance-associated protein gene. *Science* **271**: 1126–1128
Paulusma C, Kool M, Bosma PJ, Scheffer GL, ter Borg F, Scheper RJ, Borst P, Baas F and Oude Elferink RPJ (1997) A mutation in the human cMOAT gene causes the Dubin-Johnson syndrome. *Hepatology* **25**: 1539–1542
Paulusma CC, van Geer MA, Evers R, Heijn M, Ottenhoff R, Borst P and Oude Elferink RPJ (1999) Canalicular multispecfic organic anion transporter/multidrug resistance protein 2 mediates low-affinity transport of reduced glutathione. *Biochem J* **338**: 393–401
Rappa J, Lorico A, Flavell RA and Sartorelli AC (1997) Evidence that the multidrug resistance protein (MRP) functions as a co-transporter of glutathione and natural product toxins. *Cancer Res* **57**: 5232–5237
Taguchi Y, Yoshida A, Takada Y, Komano T and Ueda K (1997) Anti-cancer drugs and glutathione stimulate vanadate-induced trapping of nucleotide in multidrug resistance-associated protein (MRP). *FEBS Lett* **401**: 11–14
Tew KD (1994) Glutathione-associated enzymes in anticancer drug resistance. *Cancer Res* **54**: 4313–4320
Tietze F (1969) Enzymic method for quantitative determination of nanogram amounts of total and oxidized glutathione: applications to mammalian blood and other tissues. *Anal Biochem* **27**: 502–522
Versantvoort CHM, Broxterman HJ, Bagrij T, Scheper RJ and Twentyman PR (1995) Regulation by glutathione of drug transport in multidrug resistant human lung tumour cell lines overexpressing *MRP*. *Br J Cancer* **72**: 82–89
Wielinga PR, de Waal E, Westerhoff HV and Lankelma J (1999) In vitro transepithelial drug transport by on-line measurement: cellular control of paracellular and transcellular transport. *J Pharm Sci* **88**: 1340–1347
Zaman GJ, Flens MJ, van Leusden MR, de Haas M, Mulder HS, Lankelma J, Pinedo HM, Scheper RJ, Baas F, Broxterman HJ and Borst P (1994) The multidrug resistance-associated protein MRP is a plasma membrane efflux pump. *Proc Natl Acad Sci USA* **91**: 8822–8826
Zaman GJR, Lankelma J, van Tellingen O, Beijnen J, Dekker H, Paulusma CC, Oude Elferink RPJ, Baas F and Borst P (1995) Role of glutathione in the export of compounds from cells by the multidrug resistance-associated protein. *Proc Natl Acad Sci USA* **92**: 7690–7694

© 2000 Cancer Research Campaign 
